# Barriers to recognition of out-of-hospital cardiac arrest during emergency medical calls: a qualitative inductive thematic analysis

**DOI:** 10.1186/s13049-015-0149-4

**Published:** 2015-09-17

**Authors:** David Alfsen, Thea Palsgaard Møller, Ingrid Egerod, Freddy K. Lippert

**Affiliations:** Emergency Medical Services Copenhagen, The Capital Region of Denmark, Telegrafvej 5, 2750 Ballerup, Denmark; University of Copenhagen, Faculty of Health and Medical Sciences, 2200 Copenhagen N, Denmark; Rigshospitalet, Trauma Centre, HOC 3193, 2100 Copenhagen Ø, Denmark

## Abstract

**Background:**

The chance of surviving out-of-hospital cardiac arrest (OHCA) depends on early and correct recognition of cardiac arrest by the emergency medical dispatcher during the emergency call. When cardiac arrest is identified, telephone guided cardiopulmonary resuscitation (CPR) and referral to an automated external defibrillator should be initiated. Previous studies have investigated barriers to recognition of OHCA, and found the caller’s description of sign of life, the type of caller, caller’s emotional state, an inadequate dialogue during the emergency call, and patient’s agonal breathing as influential factors. Though many of these factors are included in the algorithms used by medical dispatchers, many OHCA still remain not recognised. Qualitative studies investigating the communication between the caller and dispatcher are very scarce. There is a lack of knowledge about what influences the dispatchers’ recognition of OHCA, focusing on the communication during the emergency call.

The purpose of this study is to identify factors affecting medical dispatchers’ recognition of OHCA during emergency calls in a qualitative analysis of calls.

**Methods:**

An investigator triangulated inductive thematic analysis of recordings of out-of-hospital cardiac arrest emergency calls from December 2012. Participants were the callers (bystanders) and the emergency medical dispatchers. Data were analysed using a hermeneutic approach.

**Results:**

Based on the concept of data saturation, 13 recordings of not recognised cardiac arrest and 8 recordings of recognised cardiac arrests were analysed. Three main themes, six subthemes and an embedded theme emerged from the analysis: caller’s physical distance (caller near patient, caller not near patient), caller’s emotional distance (keeping calm, losing control), caller is a healthcare professional (responsibility is handed over to the caller, caller assumes responsibility), and the embedded theme: caller assesses the patient.

**Conclusion:**

The physical and emotional proximity of the caller (bystander) as well as the caller’s professional background affect the dispatcher’s chances of correct recognition and handling of cardiac arrest. The dispatcher should acknowledge the triple roles of conducting patient assessment, instructing the caller, and reassuring the emotionally affected caller.

## Background

The chance of surviving from out-of-hospital cardiac arrest (OHCA) is highly associated with emergency medical dispatchers’ recognition of the condition during emergency calls, early bystander cardiopulmonary resuscitation (CPR), and early defibrillation [1–4]. It is essential that the emergency medical dispatcher (EMD) recognises OHCA, so telephone assisted CPR (tCPR) and referral to an automated external defibrillator (AED) can be initiated. Earlier studies have investigated barriers to recognition of OHCA, and found the caller’s description of signs of life, the type of caller, caller’s emotional state, inadequate dialogue during the emergency call, and patient’s agonal breathing as influential factors. Moreover, successful recognition of OHCA is associated with an assessment of the patient’s consciousness, breathing pattern, and facial colour [5–9]. Many of these factors are included in the algorithms used by medical dispatchers, but still, not all OHCA’s are recognised [10–19]. A better understanding of factors leading to successful recognition of OHCA has the potential to increase the proportion of bystander CPR, and thereby improve survival rate [20]. Qualitative studies investigating recognition of OHCA, focusing on the communication between caller and dispatcher are needed. The aim of this study was to identify factors affecting medical dispatchers’ recognition of OHCA during emergency calls in the Emergency Medical Services (EMS), Copenhagen using a qualitative analysis.

## Methods

### Setting

**Fig. 1:**
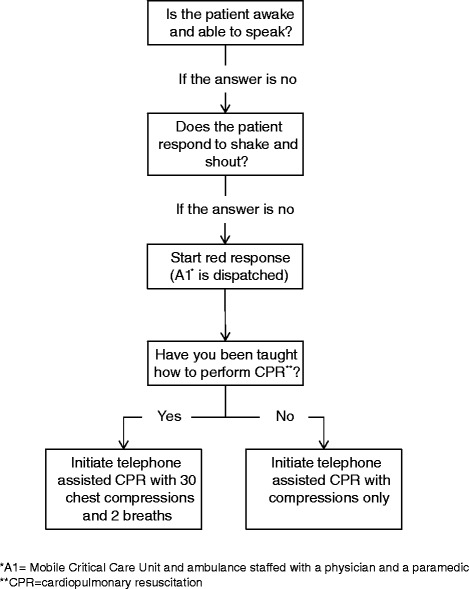
Initial steps in the algorithm for dispatch assisted telephone CPR

The study took place at the Emergency Medical Dispatch Center (EMDC) in Copenhagen, Denmark serving a population of 1.7 million and responding to about 110.000 emergency calls annually. In Denmark, there is a single emergency phone number (1-1-2) leading to an emergency call center. In case of a medical problem, the caller is re-directed to an EMDC that responds to the call by activating the appropriate response. The medical dispatchers are specially trained registered nurses and paramedics who are supported by a criteria-based, nationwide priority tool (Danish Index for Emergency Care) [21]. For each call, the system recommends a response according to contact cause and urgency of the condition. The algorithm is described in Fig. 1, and the response types are described in Table 1.

**Table 1:** Emergency response types

Emergency (lights and siren)	Urgent (no lights and siren)
A1: Mobile Critical Care Unit with physician and an ambulance staffed with paramedics	B1: Any ambulance (lights and siren if necessary)
A2: Ambulance with paramedics	B2: Ambulance with paramedics
A3: Ambulance with emergency medical technicians	B3: Ambulance with emergency medical technicians

In each emergency call, the dispatcher starts by asking the caller “How can I help you?” and then assesses the patient’s consciousness and breathing pattern. If an OHCA is suspected, the dispatcher provides telephone assisted CPR to the bystander and refers to the nearest AED. In case of suspected OHCA, a high priority response including an emergency physician is dispatched.

### Study design

An inductive qualitative approach was chosen, using recordings of emergency calls including recognised and not recognised OHCA as the source of data. The participants were callers (bystanders) and medical dispatchers. The goal of qualitative research is to understand and describe a phenomenon, including its definition, terminology and relation to other phenomena [22, 23]. Detailed description of the caller-dispatcher conversation during emergency calls paves the way to a better understanding of why OHCA is or is not recognised during the emergency call. The study was conducted in two steps: 1) sorting the recordings according to recognised and not recognised OHCA, and 2) analysing the recordings and identifying factors related to recognised and unrecognised OHCA.

### Preconceptions of the investigators

An active and deliberate relationship to their preconception or potential prejudices can aid the investigators to learn from the data [22]. In the present study, the team of investigators analysing data consisted of a registered nurse (DA), a physician (TPM), a professor in clinical nursing (IE), and the medical director of EMS Copenhagen (FKL). The specific knowledge of OHCA and EMS systems were paramount to the sorting of data items, while the difference in experience and interdisciplinary knowledge among team members facilitated critical reflection and reduced blind spots. The use of investigator triangulation strengthens the design by supplementing and challenging the investigators’ statements during the process of analysis [22, 24].

### Sampling strategy

The sample was collected in December 2012 (31 days) and the sampling strategy was criterion-based selection [22]. The calls during December 2012 were chosen because they were the most recent calls available. The criterion for selection were all calls regarding sudden unexpected cardiac arrest. Cardiac arrest due to trauma, terminal disease and patients with definite signs of death (head separated from body, rigor mortis, incineration etc.) were excluded. The OHCA calls were identified by merging databases containing mobile critical care unit charts and EMDC data. OHCA was considered as recognised if CPR was encouraged, guided, or conducted at any time during the call. Sample size was determined by saturation of data, which means that inclusion of calls continued until information became redundant, and the themes appeared robust [22].

### Analysis

Thematic analysis as described by Braun & Clarke was used, which identifies, analyses, and describes patterns in data [24]. The method is not bound by any specific theory, and does not put any specific demands on the data collection method. This pragmatic methodology lends itself for this study, because of the exploratory focus. The process of thematic analysis progresses through six phases: Phase 1: Familiarisation with data, phase 2: generating initial codes, phase 3: searching for themes, phase 4: reviewing themes, phase 5 defining and naming themes, and phase 6: producing the report. Analysis continued until saturation, viz. when information became redundant. Recordings were transcribed by simple orthographic method [24]. During analysis the recordings were played as a supplement to transcribed data in order to exploit contextual nuances [22, 24].

### Ethical considerations

The study was approved by the local ethics committee, ref. no. H-2-2014-FSP19, the Danish data protection agency, ref. no. HEH-2013-012, and I-suite no. 02112.

## Results

We included 21 emergency calls in the study; 8 with recognised OHCA and 13 with not recognised OHCA. Tables 2 and 3 show an identification number for each call, patient age and gender, the caller’s relation to the patient, the caller’s proximity to the patient and the assigned response to the emergency call for recognized and not recognised OHCA, respectively.

**Table 2:** Characteristics for emergency calls concerning recognised OHCA

Patient no.	Patient age	Patient gender	Caller's relation to patient	Caller' s proximity to patient	Response	Emergency physician treatment
1	40	Female	Spouse	With patient	A1	Resuscitation success
2	47	Male	Friend	With patient	A1	Resuscitation success
3	64	Female	Son in law	With patient	A1	No treatment
4	74	Male	Nursing home staff^a^	With patient	A1	Resuscitation success
5	75	Male	Spouse	Near patient	A1	Resuscitation success
6	81	Male	Spouse	With patient	A1	Resuscitation failed
7	83	Female	Nurse^a^	With patient	A1	No treatment
8	84	Male	Nursing home staff^a^	With patient	A1	No treatment

Patients: 5 male, 3 female, median age 74 years (40-84); caller: 3 healthcare professionals^a^, and 5 non-healthcare professionals. With patient: The caller can see the patient. Near patient: The caller cannot see the patient, but communicate with person with a person together with the patient . Resuscitation success: CPR successful. Resuscitation failed: CPR attempted, but unsuccessful. No treatment: CPR not attempted. A1 = Mobile Critical Care Unit and ambulance staffed with paramedic

**Table 3:** Characteristics for emergency calls concerning not recognised OHCA

Patient no.	Patient age	Patient gender	Caller's relation to patient	Caller's distance to patient	Response	Emergency physician treatment
9	32	Male	Mother	With patient	A3	Resuscitation success
10	49	Female	Spouse	With patient	A2	Resuscitation failed
11	59	Female	Neighbour	With patient	A2	Resuscitation failed
12	68	Male	Nursing home nurse^a^	With patient	A1	Resuscitation failed
13	71	Male	Daughter in law	Not at scene	A3	Resuscitation failed
14	74	Male	Spouse	With patient	A1	Resuscitation failed
15	77	Female	General Practitioner^a^	Not at scene	A3	No treatment
16	80	Female	Spouse	With patient	A1	Resuscitation failed
17	80	Male	Son in law	With patient	A1	Resuscitation failed
18	83	Female	Daughter	With patient	A3	No treatment
19	88	Female	Nursing home staff^a^	Near patient	A2	No treatment
20	94	Male	Spouse	Near patient	B2	No treatment
21	98	Male	Nursing home staff^a^	With patient	A1	Resuscitation failed

Patients: 6 male, 7 female, median age 74 years (32-98); Caller: 4 healthcare professionals^a^ and 9 non-healthcare professionals. With patient: The caller can see the patient. Near patient: The caller cannot see the patient, but communicate with a person together with the patient. Not at scene: Caller in different location from the patient. Resuscitation success: CPR successful. Resuscitation failed: CPR attempted, but unsuccessful. No treatment: CPR not attempted. ^a^Indicates healthcare professional. A1 = Mobile Critical Care Unit and ambulance staffed with paramedic, A2 = Ambulance staffed with a paramedic, A3 = Basic life support ambulance, B2 = Ambulance staffed with a paramedic, no lights and siren

### Themes

Three themes emerged during analysis (Table 4): caller’s physical distance (caller near patient, caller not near patient), caller’s emotional distance (keeping calm, losing control), caller is a healthcare professional (responsibility is handed over to the caller, caller assumes responsibility), and one embedded theme: Caller assesses the patient.

**Table 4:** Themes and sub-themes regarding factors affecting dispatcher's recognition of OHCA during emergency calls

Themes	Sub-themes	Embedded theme
Caller's physical distance	Caller near the patient	Caller assesses the patient
	Caller not near the patient	
Caller's emotional distance	Keeping calm	
	Losing control	
Caller is a healthcare professional	Responsibility is handed over to the caller	
	Caller assumes responsibility	

### Caller’s physical distance

#### Caller near patient

In all recognised OHCA calls, the caller was in close proximity to the patient. This enabled better communication and assessment, and the medical dispatcher was able to react quickly to information and initiate the EMD algorithm systematically with little or no interruption. If the caller was with a patient and able to describe abnormal breathing, OHCA was recognised at an early stage. During some calls the callers presented a patient with normal breathing, which changed the structure of the conversation. The dispatchers then abandoned the algorithm in favour of providing first aid, and thereby missed the provision of tCPR. If the caller stated later that the patient was deteriorating, the algorithm was applied again. This was evident in several calls where the caller interrupted the dispatcher, to describe the new situation. In the following quotation, the caller initially set the scene by presenting a respiratory problem. The dispatcher was able to explore the situation and apply the algorithm.

*[Patient 4] Dispatcher: “Hello this is the nurse from the dispatch centre”. Caller: “I’m calling from [nursing home]. I have a resident who has fallen and now he has trouble breathing; he is turning blue”. Dispatcher: “He is turning blue?” Caller: “Yes”. Dispatcher: “He is awake, isn’t he?” Caller: “More or less; no he is not”. Dispatcher: “He is not responsive?” Caller: “No, [calling name of patient]”. Dispatcher: “Is he breathing?” Caller: “Well … [pause]”. Dispatcher: “I guess not. Then you should start CPR”.*

#### Caller not near patient

As soon as it was clear to the dispatcher that the caller was not present at the scene, the algorithm from the EMD was disregarded, and a response was dispatched based on the caller’s description of the patient’s condition. In cases where the caller was closer to the patient, but not in the same room, the dispatcher had to ask the caller to go to the patient and assess the patient. These situations seemed particularly challenging when the caller was emotionally affected by the situation. The dispatcher had a double role of calming the caller while gathering relevant information. In the following the caller is calling on behalf of his mother in another part of the country.

*[Patient 13] Caller: “Yes, hello. You are speaking with [name]. My mother has just called, that her husband has collapsed. A couple of months ago he had an angioplasty”. Dispatcher: “Yes”. Caller: “and he has fainted and is unresponsive”. Dispatcher: “I see”. Caller: “She [mother] isn’t well, has seizures and …” Dispatcher: “Is it your mother or him?” Caller: “Him. My mother is so weak that she apparently isn’t able to call [mother’s location]. I am in [caller’s location] and she lives at [mother’s location]”.*

#### Caller assesses the patient

If the caller was close to the patient and able to make an assessment, this was reported to the dispatcher. In most of the recordings, the patient’s problem was initially related to respiratory failure or loss of consciousness. The dispatcher explored these avenues using the algorithm, but if the caller stated that breathing was present, the algorithm for cardiac arrest was abandoned. In some cases, the dispatcher continued to obtain a patient history without recognizing the arrest until the arrival of ambulance. The following quote illustrates a situation, where the caller believed the patient was breathing and the dispatcher as a consequence abandoned the algorithm.

*[Patient 17] Dispatcher: “Okay, but is he unconscious and not reacting to pain?” Caller: “(…), well he is breathing (…), but not reacting”. Dispatcher: “Then you have to make sure he is breathing, and that he hasn’t arrested (…) try to see if his chest is moving.” Caller: “It is.” Dispatcher: “It is, and is it moving properly?” Caller: “I would think so (…)”.*

### Caller’s emotional distance

#### Keeping calm

In calls with recognised OHCA, the dispatcher was able to provide instructions to the caller in a calm, clear and direct way, and the caller followed the instructions. The caller became an active partner in patient assessment adding to the success of the call. The dispatcher was able to establish a good communication with the caller and systematically follow the algorithm. The following is an example of direct and good communication.

*[Patient 2] Caller: “Come on, again. Breathe. It's as if he's dropping his tongue, like he can’t hold it himself.” Dispatcher: “No, but if you hold the jaw, pulling the” (caller interrupts) Caller: “I have two fingers inside the mouth, holding the tongue.” Dispatcher: “You pull the jaw forward” (speaking slowly and loud). Caller: “Come on. Hell, yes there it was” (loud and strained, then relieved). Dispatcher: “That's great, and how is his colour now, any better?” Caller: “No, he is still completely blue and completely dead” (calm and clear). Dispatcher: “Try to put him onto his back. You have to start CPR."*

#### Losing control

An emotionally affected caller had more trouble recognising OHCA. The dispatcher assessed the condition of the caller listening for signs of crying or talking. In not recognised OHCA the dispatcher might choose to calm the caller before continuing. In some cases, the dispatcher became emotionally affected in response to the caller’s distress. In the following, the dispatcher tried to maintain patient assessment despite the distress of the caller.

*[Patient 20] Dispatcher: “You need to pay attention; I’m going to ask you some questions. I already sent an ambulance, okay?” Caller: “Yes. (…).“ Dispatcher: “Is he breathing, your husband? “ Caller: “Yes, I hope (trembling voice)“. Dispatcher: “What is the colour of his face?” (Interrupting) Caller: “Well I can’t tell you more, now I have to go. You have to come (loud and determined)“. (Interrupting) Dispatcher: “No, go and look at his face” (caller hangs up).*

### Caller is a healthcare professional

#### Responsibility is handed over to the caller

When the dispatcher recognised the caller as a healthcare professional, the roles were reversed; the caller was given responsibility and took action while the dispatcher had a counselling role. In recordings with recognised OHCA, the caller recognized the warning signs. In the following the caller identified OHCA and the dispatcher initiated tCPR.

*[Patient 8] Dispatcher: “Uhm, does he have any heart disease or pulmonary diseases?” Caller: “Yes, COPD and something with his heart”. Dispatcher: “Okay, it can be either one or the other we are dealing with.” Caller: “Yes, I just came here, because, uhm, I had to check his medications and will initiate a resuscitation attempt because he stopped breathing (…).” Dispatcher: “Do you have a defibrillator?” Caller: “No (…).” Dispatcher: “How’s the breathing now?” Caller: “Right now he is pausing and the pauses are getting longer”. Dispatcher: “How about the chest, does it move up and down when he breathes?” Caller: “Uhm, right now there is no breathing.” Dispatcher: “Then you might want to start CPR”.*

#### Caller assumes responsibility

In some calls with not recognised OHCA, the dispatcher indirectly transferred the responsibility to the caller, if a healthcare professional. This phenomenon was characterised by a relaxed collegial atmosphere, where the algorithm for cardiac arrest was abandoned in favour of more informal assessment and the use of ambiguous terms, e.g. “fairly unconscious”.

*[Patient 21] Caller: “He is fairly unconscious at the moment (…).” Dispatcher: “Is he breathing now?” Caller: “He is breathing now and then, and then he stops, uhm, it sound like fluids are accumulating (…)”. Dispatcher: ”Okay, and now you are telling me that he is unconscious?” Caller: “He is unconscious now, yes.” Dispatcher: “Yes, okay. And breathing is slower…” Caller: “Slower breathing (…)” Dispatcher: “Good, then that’s settled, bye.”*

## Discussion

The aim of this study was to identify factors affecting medical dispatchers’ recognition of OHCA during emergency calls. The main findings included three themes: “caller’s physical distance”, “caller’s emotional distance”, and “caller is a healthcare professional”. The dispatchers often had a triple role; trying to obtain patient information, instructing the caller, and calming the caller. This triple role was evident in two subthemes: “Caller not near the patient” and “losing control”.

In our study, the dispatcher did not strictly use the cardiac arrest algorithm if the caller was not near the patient. This finding is supported by several studies that have demonstrated the distance between caller and patient being a potential barrier to the recognition of OHCA [16, 25–27]. One study including interviews with 10 dispatchers showed that tCPR was unlikely if the caller was not at the scene [28]. The same study showed that the emotional state of the caller influenced initiation of tCPR. In accordance with our study, several studies have shown how the emotional state of the caller affected OHCA recognition and precluded tCPR [27, 29–31].

Our study has shown that it is risky if the dispatcher disregards the algorithm, even if the situation appears under control. Similar results have been reported in a recent study showing that the main barriers to recognition of OHCA were absence or incomplete assessment of breathing and the presence of agonal breathing [32].

In our sub-theme “losing control” we found that the emotional state of the caller could influence the dispatcher. We also observed the inherent emotional asymmetry between caller and dispatcher, as the caller was typically experiencing a unique and devastating situation, whereas the dispatcher was doing "business as usual". In the sub-theme “losing control” the asymmetry was evident, but in some cases the dispatchers also got close to losing control. If, on the other hand, the caller was calm, or reassured by the dispatcher, the asymmetry was replaced by a constructive and information rich conversation.

The characteristics of the callers, as illustrated in Tables 2 and 3, showed that all callers in the group that recognised OHCA were in close proximity to the patient. This was supported in the theme “caller’s physical distance”. Importantly, more patients were successfully resuscitated when OHCA was recognised, potentially increasing patients’ chances of survival [20].

Surprisingly, the theme “caller is a healthcare professional”, showed that the caller’s profession did not necessarily increase the chances of OHCA recognition. Some of these callers were nursing home staff, and might not be as updated in emergency care and cardiac arrest, as presumed by the dispatcher. When the dispatcher let the caller take command and control, the cardiac arrest algorithm was abandoned with poor results. Dispatchers’ use of the algorithm is a modifiable factor, and it should be emphasized that the algorithm must be used even if the caller is a healthcare professional. Other studies have described the phenomenon of significantly lower OHCA recognition in professionals than non-professional bystanders, and less use of the algorithm among healthcare professionals [12].

In our embedded theme “caller assesses the patient” we found that the dispatcher abandoned the cardiac arrest algorithm if the caller identified breathing or signs of life. Other studies have found that the description of signs of life is one of the most significant factors leading to failed recognition of OHCA [16]. Reasons for this might be that the patient does not have cardiac arrest before the arrival of the ambulance, or especially, that the patient has agonal breathing, which is present in 40 % of OHCA’s, and is known to delay or impede recognition of OHCA [16, 28, 33–36]. Another reason could be bystanders’ difficulties in assessing breathing and consciousness, potentially leading to incorrect information passed to the dispatcher [37]. An implication of this knowledge could be to acknowledge the dispatcher, caller and other bystanders as a “first resuscitation team” working towards the common goal of recognising OHCA [38].

Inductive thematic analysis has been a strong methodological tool to meet the purpose of this study. There are limitations, which should be addressed. Most importantly, we cannot be sure if calls identified as not recognised OHCA, occurred prior to the call or after termination of the call before arrival of the ambulance. It can be argued, however, that OHCA was imminent and therefore still essential to recognise. To support the credibility of the study, investigator triangulation was used. Generalizability was increased by external studies showing similar results and by other dispatchers in Europe using the same EMD system. Reliability was achieved by saturation of data.

## Conclusion

The study shows that the caller has a central role in recognising and acting upon OHCA during emergency calls. The physical and emotional proximity of the caller (bystander) as well as the caller's professional background affect the dispatcher's chances of correct recognition and handling of cardiac arrest. The dispatcher should acknowledge the triple roles of conducting patient assessment, instructing the caller, and reassuring the emotionally affected caller.
